# Feline Panleukopenia Virus With G299E Substitution in the VP2 Protein First Identified From a Captive Giant Panda in China

**DOI:** 10.3389/fcimb.2021.820144

**Published:** 2022-02-07

**Authors:** Shushuai Yi, Songrui Liu, Xianyong Meng, Pei Huang, Zengguo Cao, Hongli Jin, Jianzhong Wang, Guixue Hu, Jingchao Lan, Dongsheng Zhang, Yuwei Gao, Hualei Wang, Nan Li, Na Feng, Rong Hou, Songtao Yang, Xianzhu Xia

**Affiliations:** ^1^Changchun Veterinary Research Institute, Chinese Academy of Agricultural Sciences, Changchun, China; ^2^College of Animal Science and Technology, Jilin Agricultural Science and Technology University, Jilin, China; ^3^Chengdu Research Base of Giant Panda Breeding, Sichuan Key Laboratory of Conservation Biology for Endangered Wildlife, Sichuan Academy of Giant Panda, Chengdu, China; ^4^College of Veterinary Medicine/College of Animal Science and Technology, Jilin Agricultural University, Changchun, China; ^5^Key Laboratory of Zoonosis Research, Ministry of Education, College of Veterinary Medicine, Jilin University, Changchun, China

**Keywords:** FPV, giant pandas, G299E, VP2 protein, molecular characterization

## Abstract

A feline panleukopenia virus (FPV), Giant panda/CD/2018, was isolated from a captive giant panda with mild diarrhea in 2018 in Chengdu, China, and further identified *via* indirect immunofluorescence assay (IFA), transmission electron microscopy (TEM) observation, and genetic analysis. Phylogenetic analysis based on the complete VP2 nucleotide sequences showed that it shared high homology with Chinese FPV isolates and grouped within FPV cluster 1. One unique substitution Gly(G)299Glu(E) in the capsid protein VP2 was first identified with Giant panda/CD/2018. The presence of the G299E substitution is notable as it is located on the top region of the interconnecting surface loop 3, which may be involved in controlling the host range and antigenicity of FPV. These findings first demonstrate that FPV with natural point mutation G299E in the VP2 gene is prevalent in giant panda and suggest that etiological surveillance and vaccination among all giant pandas are urgently needed to protect this endangered species against FPV infection.

## Introduction

Giant panda (*Ailuropoda melanoleuca*), originating from China, is the sole strictly herbivorous bear species within the family *Ursidae*, while other members are omnivores or carnivores (Wei et al., 2012). As an endangered species listed in the International Union for the Conservation of Nature (IUCN), the giant panda is one of the flagship species of the world’s biodiversity conservation. Currently, giant pandas face many threats, including low reproductive success; the loss, degradation, and fragmentation of habitat; and the challenges of infectious disease (Kang and Li, 2019). Notably, the habitat modifications increase the risk of exposure of pandas, especially captive pandas, to more pathogens. In the past few decades, viral infections, including canine distemper virus (CDV) (Qin et al., 2010; Feng et al., 2016), canine coronavirus (CCV), canine parvovirus (CPV) (Qin et al., 2010), canine adenovirus (CAV), H1N1 influenza A virus (Li et al., 2014), and rotavirus, had threatened the health of giant pandas, and were even fatal (Hvistendahl, 2015). Hence, the etiological and serological surveillance of viral infections that threatened the health of giant pandas should be given great emphasis.

Carnivore protoparvovirus 1, a viral species of the genus *Protoparvovirus* within the family *Parvoviridae*, is a worldwide epidemic with a broad host range (Cotmore et al., 2019). According to the International Committee on Taxonomy of Viruses (ICTV) report updated in 2019 (https://talk.ictvonline.org/taxonomy/), four members were grouped and distinguished in the species Carnivore protoparvovirus 1, including feline panleukopenia virus (FPV), CPV, mink enteritis virus (MEV), and raccoon parvovirus, on the basis of host range and several key amino acid positions mutations in the VP2 gene (Mietzsch et al., 2019). Several previous reports had demonstrated that CPV are responsible for diarrhea and mortality in giant pandas (Wu et al., 1988). However, there was no abundant evidence to prove the circulation of other Carnivore protoparvovirus 1 in giant pandas, such as FPV.

In the present study, we carried out the etiological surveillance of Carnivore protoparvovirus 1 in captive giant pandas from Chengdu Research Base of Giant Panda Breeding. A unique FPV strain with Gly(G)299Glu(E) mutation on the loop 3 region of the threefold spike of VP2 protein was isolated from captive adult giant pandas with mild diarrhea.

## Methods

### Virus Isolation and Identification

To supervise the circulation of carnivore protoparvovirus 1 in giant pandas living in Chengdu, fecal samples were quarterly collected and tested for Carnivore protoparvovirus 1 as previously described (Yang et al., 2010). Briefly, viral DNA was extracted from individual fecal samples using AxyPrep Multisource Genomic DNA Miniprep Kit (CORNING, China), and then was used as template for the detection of carnivore protoparvovirus 1 with a pair of universal primers (P1, 5’-GGATGGGTGGAAATCACAGC-3’; P2, 5’-ATAACCAACCTCAGCTGGTC-3’) targeting 845 bp of the partial VP2 gene. The supernatant of parvovirus-positive sample was filter-sterilized *via* 0.22-μm filtering film. Then, the filtered supernatants were synchronously inoculated with feline kidney 81 (F81) cell and cultured at 37°C in a 5% CO_2_ incubator.

The cytopathic effect (CPE)-positive cell cultures were centrifuged at 30,000×*g* for 10 min, and then negatively stained using 0.5% phosphotungstic acid and observed by transmission electron microscopy (TEM). Furthermore, the harvested virus was further identified by indirect immunofluorescence assay (IFA) using murine monoclonal antibody (Ab) against FPV (Abcam, United Kingdom) as primary Ab and FITC-conjugated goat anti-mouse IgG H&L (Abcam, United Kingdom) as second Ab. Hemagglutination (HA) test was performed according to the protocol described previously.

### Sequencing and Phylogenetic Analysis

In order to further analyze the genetic characteristics of giant panda-derived FPV, the full-length VP2 gene were amplified using primer pairs P5 (5’-CTCGGATCCCCAATGAGTGATGGAGCAGTTCAACCAGAC-3’) and P6 (5’-AACCTCGAGCTAGGTGCTAGTTGATATGTAATAAAC-3’) previously described (Yang et al., 2010). The nucleotide and deduced amino acid sequences of the full-length VP2 gene obtained from giant panda-sourced FPV were compared with other reference carnivore protoparvovirus 1 strains listed in the GenBank database. Multiple sequences alignment was performed using Clustal W method in MEGA 7.0 software, and the percentage pairwise identities were calculated using Bioedit. Neighbor-joining (NJ) analysis was performed in MEGA 7.0 using the Kimura 2-parameter model with 1,000 bootstrap replicates. The NJ tree was visualized using Figtree v1.4.3. Phylogenetic analysis was further confirmed using Maximum likelihood (ML) method.

### Molecular Modeling of Capsid Mutations

To predict whether the mutations observed in the VP2 gene of giant panda-sourced FPV affected the overall structure of the capsid, molecular modeling of capsid mutations was performed using the mutagenesis function in PyMOL Molecular Graphics System. The crystal structure of FPV (PDB ID: 1FPV) was used for molecular modeling (Agbandje et al., 1993).

## Results

**Figure 1:**
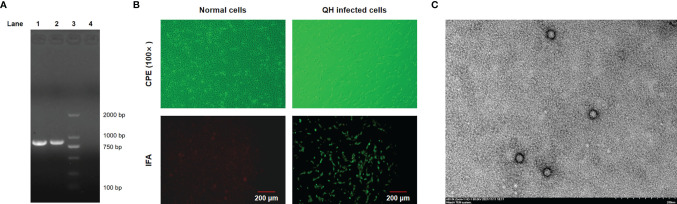
Identification of FPV Giant panda/CD/2018 through IFA, TEM, and PCR. **(A)** PCR products were analyzed by 1.5% agarose gel. Lane 1, the isolate Giant panda/CD/2018; Lane 2, FPV prototype CU-4; Lane 3, DL 2000 DNA marker; Lane 4, nucleotide-free water (negative control). **(B)** The cytopathic effect (CPE) and IFA identification. **(C)** Negative-stained transmission electron microscopy of FPV Giant panda/CD/2018 particles.

Fecal samples collected from two captive adult giant pandas with mild diarrhea (CD-1 and CD-2) in December 2018 were tested positive for carnivore protoparvovirus 1 by conventional PCR using the universal primers and showed 100% nucleotide identities with each other based on the partial VP2 gene. Fecal sample from one giant panda (CD-1) was selected for virus isolation. The typical CPE of parvovirus, characterized by cell rounding, pyknosis, disruption, and eventually necrosis, shed completely, appeared in F81 cells inoculated with the isolated virus (**Figure 1A**). A giant panda-sourced FPV isolate named Giant panda/CD/2018 was successfully obtained from fecal samples identified by PCR, IFA, and TEM. As shown in **Figure 1**, an 845-bp PCR fragment that is positive for carnivore protoparvovirus 1 was obtained from this isolate Giant panda/CD/2018, and bright green fluorescence was also observed in F81 cells infected with this isolate; in contrast, no fluorescence was found in normal cells. TEM of negative stained isolate revealed spherical virus particles with a diameter of 20–25 nm, which is in accordance with the structural characteristics of typical parvovirus. The HA assay results showed that Giant panda/CD/2018 displays hemagglutination virus properties well with a titer as high as 1:1,024.

The full-length VP2 gene of the isolate Giant panda/CD/2018 was amplified and sequenced, yielding a 1,755-bp fragment, which encodes 584 amino acids (aa). Multiple sequences alignment based on the nucleotide (nt) and deduced amino acid (aa) sequences of the VP2 gene showed that the isolate Giant panda/CD/2018 shared more than 98% nt and 97.5% aa identities with carnivore protoparvovirus 1. This isolate displayed higher nt and aa identities to FPV (99.0%–99.6% and 99.1%–99.8%) than CPV (98.4%–98.8% and 97.7%–98.2%). To determine the molecular characteristics of the isolate Giant panda/CD/2018, the critical amino acid positions of the parvovirus VP2 gene (Barrs, 2019) were analyzed and are summarized in **Table 1**. The amino acid residues at positions 80, 93, 103, 323, 564, and 568 of Giant panda/CD/2018 VP2 protein, which determined the host specificity of FPV and CPV, were identical with all FPV reference strains, further revealing that the isolate Giant panda/CD/2018 was FPV.

**Table 1:** Amino acid residue characteristics and pairwise identity in the VP2 protein of giant panda-derived FPV compared with other related parvovirus strains.

Isolate	Amino acid residues														Nt/aa identity (%)^#^	Antigenic type
	80	85	87	93	101	103	232	299	300	305	323	426	564	568		
Giant panda/CD/2018 (MZ322607)	K	N	M	K	T	V	V	**E**	A	D	D	N	N	A	–	FPV
CD-1 (fecal sample)	K	N	M	K	T	V	V	**E**	A	D	D	N	N	A	100/100	FPV
CD-2 (fecal sample)	K	N	M	K	T	V	V	**E**	A	D	D	N	N	A	100/100	FPV
CU-4 (M38246, USA)	K	N	M	K	I	V	V	G	A	D	D	N	N	A	99.2/99.6	FPV
Purevax Merial vaccine (EU498680)	K	N	M	K	I	V	I	G	A	D	D	N	N	A	99.3/99.3	FPV
Felocell Pfizer vaccine (EU498681)	K	N	M	K	T	V	I	G	A	D	D	N	N	A	99.3/99.4	FPV
TU-2 (AB000066, Japan)	K	I	M	K	T	V	V	G	A	D	D	N	N	A	99.2/99.6	FPV
Changc2007 (FJ936171, China)	K	I	M	K	T	V	I	G	A	D	D	N	N	A	99.6/99.4	FPV
CPV-b (M38245, USA)	R	N	M	N	I	A	I	G	A	D	N	N	S	G	98.8/98.2	CPV-2
CPV-15 (M24003, USA)	R	N	L	N	T	A	I	G	G	Y	N	N	S	G	98.6/97.9	CPV-2a
CPV-436 (AY742955, USA)	R	N	L	N	T	A	I	G	G	Y	N	D	S	G	98.4/97.7	CPV-2b
695 (AF401519, Italy)	R	N	L	N	T	A	I	G	G	Y	N	E	S	G	98.5/97.7	CPV-2c

The amino acid positions are referred to the complete VP2 protein of FPV prototype (CU-4, M38246). Bold text indicates the unique amino acid substitutions for giant panda-sourced FPV isolated in this study. ^#^Nucleotide/amino acid identity in the VP2 gene compared to giant panda-sourced FPV.

**Figure 2:**
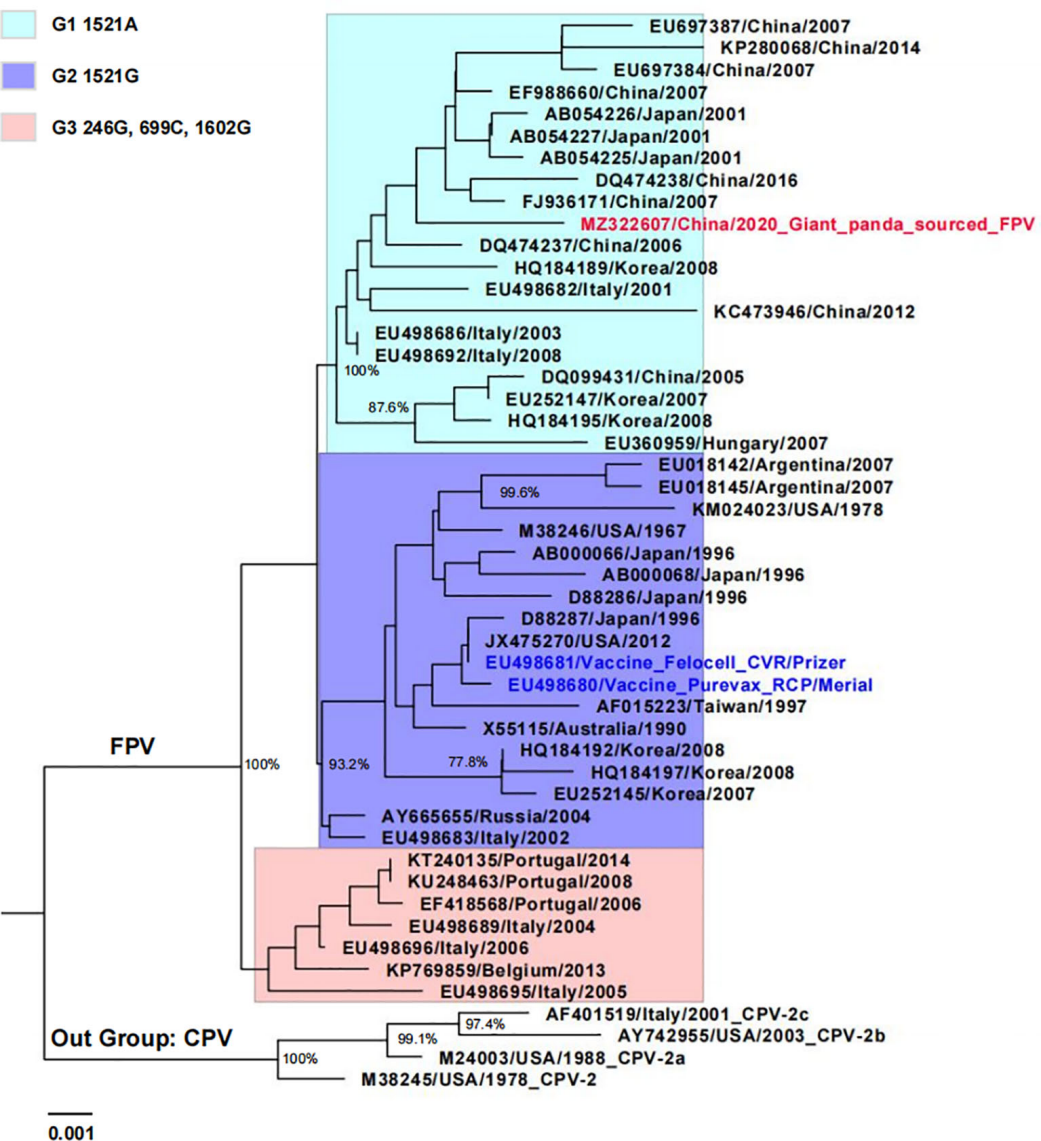
Phylogenetic tree of giant panda-derived FPV isolate compared with other related parvovirus strains obtained from the GenBank database based on the full-length VP2 nucleotide sequences. The tree was generated using the neighbor-joining method in MEGA 7.0 with 1,000 bootstrap values and visualized using Figtree. Only bootstrap values >70% were displayed above the tree branches. Horizontal branch lengths are proportional to genetic distances. Scale bars indicate nucleotide substitutions per site. Red text indicates giant panda-sourced FPV isolated in the present study; blue text indicates FPV vaccine strain; FPV cluster 1 (G1), 2 (G2), and 3 (G3) are shown with different background colors.

The NJ tree based on the VP2 nucleotide sequences showed that Giant panda/CD/2018 was more closely related to 44 FPV representative strains isolated from different countries, clustering within the group FPV (**Figure 2**). Furthermore, all FPV strains were classified within three clusters according to the nucleotide mutations: G1 (1521A), G2 (1521G), and G3 (246G, 699C, and 1602G). The isolate Giant panda/CD/2018 was located in FPV G1, which consisted of all Chinese isolates and partially from Japan, South Korea, and Italy, and formed a monophyletic branch.

**Figure 3:**
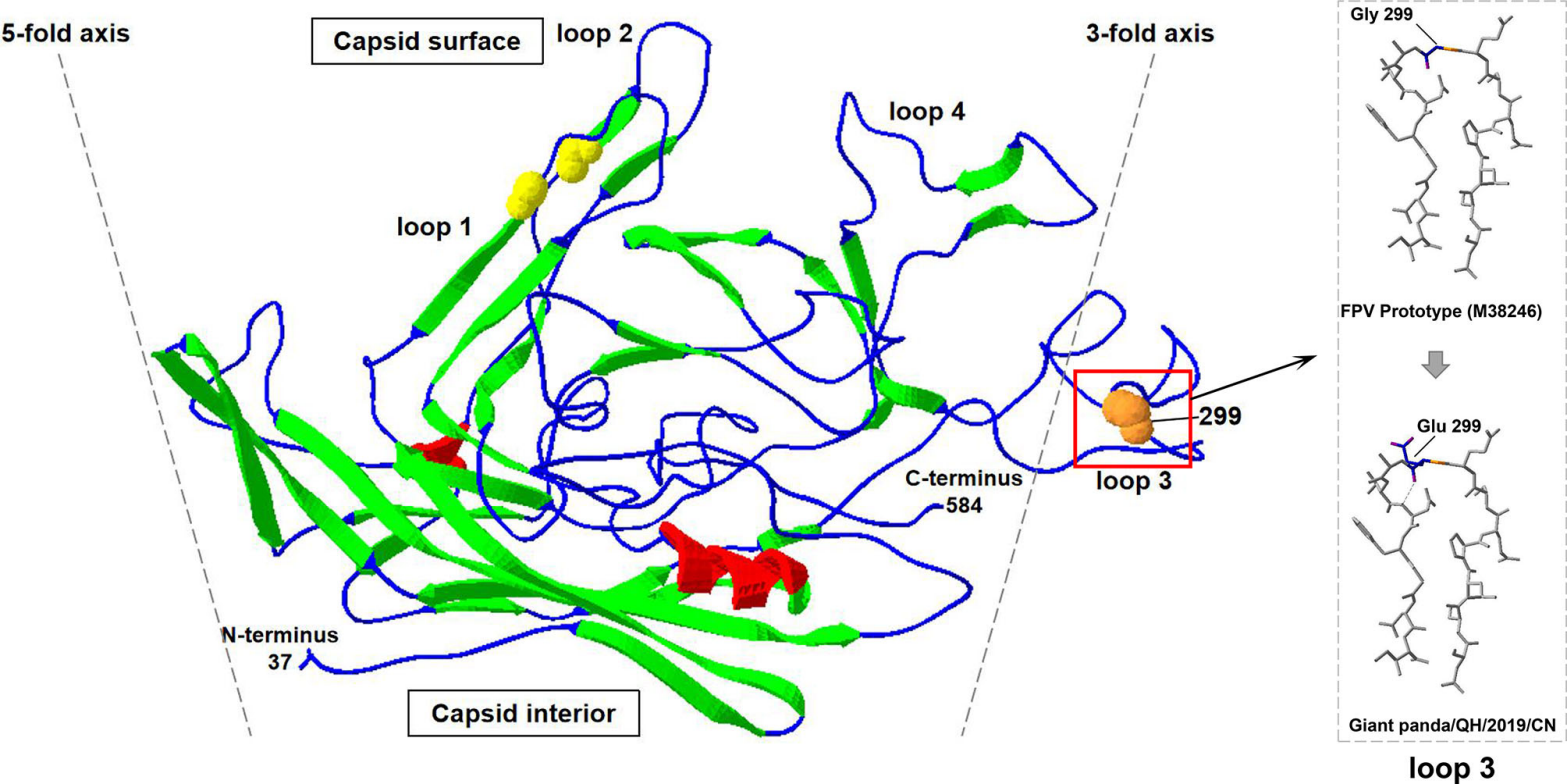
The cartoon ribbon diagram of a VP2 capsid monomer of FPV (PDB ID: 1FPV) when viewed tangentially to the surface of the virus. The β-strands, α-helix, and interconnecting surface loop are indicated by green, red, and blue. Spheres show the positions of the natural (orange) mutations of the isolate Giant panda/CD/2018. Loop 3 that contained amino acid mutation is labeled *via* black boxes, and is magnified in dotted boxes.

Furthermore, compared to FPV prototype CU-4 (GenBank accession number M38246), five nucleotide changes at positions 302 (T→C), 546 (T→C), 896 (G→A), 1,149 (A→G), and 1,747 (T→C) were observed in the VP2 gene of Giant panda/CD/2018, resulting in three synonymous mutations at residues 182, 383, and 583 and two non-synonymous mutations at residues 101 and 299 (**Table 1**). The amino acid mutation Ile(I)101Thr(T) has been reported in many FPV strains isolated from China, Japan, and other countries, while the mutation G299E was unique for the isolate Giant panda/CD/2018, which was not found in all known FPV and CPV isolates. Moreover, the complete VP2 gene of two parvovirus-positive samples CD-1 and CD-2 were also sequenced, and showed 100% nucleotide and amino acid identities with the isolate Giant panda/CD/2018, with the same G299E point mutation in the VP2 gene, which further determined that the mutation G299E was unique for FPV from Giant panda. The molecular structure of the VP2 monomer of Giant panda/CD/2018 was predicted according to the crystal structure of FPV (PDB ID: 1FPV) (Agbandje et al., 1993). As shown in **Figure 3**, VP2 was composed of a core eight-stranded β-barrel motif, of which some β-strands were connected *via* interconnecting surface loops. The unique mutation G299E was located on the surface-exposed part (299-300-301) of VP2 loop 3 on the shoulder of the threefold spike of the capsid, which was one of the key determinants for host range and antigenicity (Allison et al., 2016; Callaway et al., 2018).

## Discussion

Carnivore protoparvovirus 1, a worldwide epidemic parvovirus with a broad host range, can infect most of domestic or wild carnivores in the order *Carnivora*, including *canidae*, *felid*, *mustelid*, and *procyonidae*. FPV and CPV are the most common carnivore protoparvovirus 1, and have the wider host range and higher pathogenicity (Barrs, 2019). At present, FPV have been reported to infect domestic cats, tiger (Duarte et al., 2009), lions (Foley et al., 2013), linsang (Inthong et al., 2019), and monkey (Yang et al., 2010); however, to our knowledge, it has not been previously reported in giant panda. Here, the existence of FPV infection in giant pandas was firstly demonstrated with the clinical symptom of mild diarrhea. Undoubtedly, it will be helpful for the pathogenicity study and vaccine development for FPV infection in giant pandas.

For wild and/or captive carnivores, FPV transmission predominantly occurs *via* indirect contact with fomites from domestic cats. In Portugal, Duarte et al. described two cases of fatal infection caused by FPV in a white tiger and an African lion at the Lisbon zoo, and demonstrated that stray cats were the source of infection *via* sequence analysis (Duarte et al., 2009). In India, domestic cats were considered to be source of the circulation of FPV in leopards between 2008 and 2014 (Shetty et al., 2020). In this study, giant panda-sourced FPV isolate shared high nucleotide and deduced amino acid identities with Chinese FPV strains isolated from domestic cats based on the full-length VP2 gene. Furthermore, phylogenetic analysis showed that giant panda-sourced FPV isolate and Chinese FPV strains grouped within the same cluster (G1), which suggests that domestic or stray cats may have been the source of infection (**Figure 2**). Furthermore, there is a great possibility that giant panda keepers, as well as food and utensils that entered breeding base, carried FPV *via* contacting FPV-infected cats or excretions, undoubtedly increasing the risk of FPV transmission. Tracing the source of giant panda’s FPV will be considered in our future investigation for better prevention and control.

Most viruses showed limited ability to adapt to the new host, because the viruses cannot recognize the corresponding receptors from the host, while interspecies transmissions seem to be frequent for FPV. Evolution and variation from FPV to MEV to CPV-2 to CPV-2a to CPV-2b to CPV-2c showed the strong ability of FPV to adapt to a new host (Ikeda et al., 2002). In this study, we found a unique amino acid mutation at residue 299 (Gly→Glu) of capsid protein VP2 in giant panda-sourced FPV (**Table 1**), which was not reported in FPV or CPV that was previously isolated. This unique mutation was located on the top region of VP2 loop 3 on the shoulder of the threefold spike of the parvovirus capsid (**Figure 3**). The capsid protein of parvovirus is composed of eight β-strands connected by interconnecting surface loops (Allison et al., 2016). One bulging area of the capsid known as the threefold spike, containing epitopes involved in transferrin receptor (TfR) binding and infection, is critical in controlling both the antigenicity and host range of FPV and CPV (Parker and Parrish, 1997; Callaway et al., 2018). Residues 297–302 are located on top of loop 3 and are exposed on the surface of the capsid protein VP2. These surface-exposed residues form part of the region that consists of five polypeptide loops from three different capsid monomers, on a ridge of the threefold spike of the capsid termed the shoulder (Tsao et al., 1991). It has been demonstrated that the surface-exposed residues of loops (including residues 297, 299, 300, and 301) are not directly involved in TfR binding, but they may instead sterically interfere with TfR binding or control the structural transitions required for capsid infectivity to control the host range and antigenicity of FPV (Llamas-Saiz et al., 1996; Hafenstein et al., 2009; Allison et al., 2016). Previous studies had demonstrated that the CPV-2 infectious clone with the mutation G299E lost the ability to replicate in canine cells, revealing that 299-Glu can severely restrict the infection of CPV in canine cells (Allison et al., 2016). For FPV, the infectious clone with 299-Glu can replicate in feline and mink cells, but the variant with 299-Glu has not been found in field isolates, suggesting that 299-Glu may be deleterious in nature. In the present study, the natural 299-Glu variant was successfully isolated from giant panda and might be prevalent in this breeding base (Allison et al., 2016). We provide the first evidence that FPV with 299-Glu position mutation is present in nature. However, we cannot be certain about the origin of this mutation. On the one hand, the 299-Glu mutation of FPV may occur in giant panda to acquire the infected ability or adapt to the new host, similar to CPV-2. On the other hand, this mutation may occur in domestic cats or wild felids and then transmitted to the giant panda. Furthermore, the region 299-300-301 on loop 3 forms part of neutralizing site B of FPV and CPV, and the mutation at residue 299 may also affect the antigenicity of FPV, which will be our research focus in the future (Hafenstein et al., 2009; Mietzsch et al., 2019).

In conclusion, an FPV isolate Giant panda/CD/2018 was obtained from a captive giant panda in China, and the molecular characteristics of this isolate were analyzed. This giant panda-sourced FPV isolate has a unique glutamic acid substitution at residue 299 located on the top region of the interconnecting surface loop 3 of capsid protein VP2. We not only first determine the prevalence of FPV in giant panda, but also first describe the FPV isolate with natural G299E mutations. This giant panda-sourced FPV will be helpful for the pathogenicity study and vaccine development to preferably protect this endangered species against FPV infection.

## Data Availability Statement

The data presented in this study are deposited in the GenBank repository, accession number MZ322607.

## Author Contributions

NF, RH, STY, and XX designed the study. SSY, SL, JW, GH, and JL drafted the manuscript. XM, PH, ZC, and HJ analyzed the data. DZ, YG, and HW contributed substantially to the content and reviewed or edited the manuscript. All authors contributed to the article and approved the submitted version.

## Funding

This research was funded by the Research Project of the National Key Research and Development Plan of China (Grant no. 2017YFD0501702-6) and Chengdu Research Base of Giant Panda Breeding (2020CPB-B22) and Shanxi Special plan for the Giant panda International Cooperation Fund Project (2019-93-1).

## Conflict of Interest

The authors declare that the research was conducted in the absence of any commercial or financial relationships that could be construed as a potential conflict of interest.

## Publisher’s Note

All claims expressed in this article are solely those of the authors and do not necessarily represent those of their affiliated organizations, or those of the publisher, the editors and the reviewers. Any product that may be evaluated in this article, or claim that may be made by its manufacturer, is not guaranteed or endorsed by the publisher.
